# Supplementary material for: Adult plant resistance in maize to northern leaf spot is a feature of partial loss-of-function alleles of Hm1
Source: PLoS Pathog. 2018 Oct 17;14(10):e1007356. doi: 10.1371/journal.ppat.1007356 (PMC6205646; doi:10.1371/journal.ppat.1007356)
Supplement: S1 Fig — Sequence comparison of the Hm1 promotor from different Hm1 alleles that exhibit complete resistance (in B73, Va35, and Pr1 inbreds) against Cochliobolus carbonum race 1 with adult plant resistance (APR) manifesting Hm1A (from P8 inbred) and completely susceptible hm1 (from Pr inbred). Promotor of Hm1 was almost similar for the first -200 bp from the transcription start site (TSS) for all Hm1 alleles. From -200 bp onwards, the promotor sequence of Hm1A differed from CCR1-resistant Hm1B73 but similar to Hm1 alleles from CCR1-resistant inbred Va35 and Pr1, and CCR1-susceptible Pr. (PDF) [file ppat.1007356.s001.pdf]

S1 Fig. Promotor comparison of different *Hm1* alleles. Sequence comparison of the *Hm1* promotor from different *Hm1* alleles that exhibit complete resistance (in B73, Va35, and Pr1 inbreds) against *Cochliobolus carbonum* race 1 with adult plant resistance (APR) manifesting $Hm1^A$ (from P8 inbred) and completely susceptible *hm1* (from Pr inbred). Promotor of *Hm1* was almost similar for the first -200 bp from the transcription start site (TSS) for all *Hm1* alleles. From -200 bp onwards, the promotor sequence of $Hm1^A$ differed from CCR1-resistant $Hm1^{B73}$ but similar to *Hm1* alleles from CCR1-resistant inbred Va35 and Pr1, and CCR1-susceptible Pr.

```
Hm1-B73       -------TTAATGAAGTTATCGATGTTTGTATATTTCACGACTATAACCTTCACCTCTGA
Hm1A          -TTGGACTTAATGACGTGCCAGTCGAATGGCT----------TCCATCCTTCT-----G-
Hm1-Va35      ATTGGACTTAATGACGTGCCAGTCGAATGGCT----------TCCATCCTTCT-----G-
Hm1-Pr1       ATTGGACTTAATGACGTGCCAGTCGAATGGCT----------TCCATCCTTCT-----G-
hm1-Pr        ATTGGACTTAATGACGTGCCAGTCGAATGGCT----------TCCATCCTTCT-----G-
                     *******.** . .*: *::** .*          *. *:*****:     *

Hm1-B73       CCGCTAATGCTTGTTTAGACCATATTTG--------------------------------
Hm1A          ----TACTGCTAGTTTCACCAATTCTCGGGTGATGTGCGTTCCCTTGTTATGGATTTCTT
Hm1-Va35      ----TACTGCTAGTTTCACCAATTCTCGGGTGATGTGCGTTCCCTTGTTATGGATTTCTT
Hm1-Pr1       ----TACTGCTAGTTTCACCAATTCTCGGGTGATGTGCGTTCCCTTGTTATGGATTTCTT
hm1-Pr        ----TACTGCTAGTTTCACCAATTCTCGGGTGATGTGCGTTCCCTTGTTATGGATTTCTT
                  **.****:****...*.**: * *

Hm1-B73       ------------------GATGTTGTTGTAT---------TAA--AATATTAT-------
Hm1A          CATGCATGA------ATGGGTGTTGTGAGCTTTTGGGTAGTCAATCTTTTTATGTGACAA
Hm1-Va35      CATGCATGATGGTCCATGGGTGTTGTGAGCTTTTGGGTAGTCAATCTTTTTATGTGACAA
Hm1-Pr1       CATGCATGATGGTCCATGGGTGTTGTGAGCTTTTGGGTAGTCAATCTTTTTATGTGACAA
hm1-Pr        CATGCATGATGGTCCATGGGTGTTGTGAGCTTTTGGGTAGTCAATCTTTTTATGTGACAA
                                *.****** . .*         *.*  .:*:****

Hm1-B73       --GTTATTAAGGAACCATTGTTTTAGCATTGTATTCAAAATTATGAAGTT--TTTATATG
Hm1A          TCGTTCGTTGGGTGCCAAGGGTGTTTAAT-TTCTCCA--TGTATGAAGAAATTTGACTTG
Hm1-Va35      TCGTTCGTTGGGTGCCAAGGGTGTTTAAT-TTCTCCA--TGTATGAAGAAATTTGACTTG
Hm1-Pr1       TCGTTCGTTGGGTGCCAAGGGTGTTTAAT-TTCTCCA--TGTATGAAGAAATTTGACTTG
hm1-Pr        TCGTTCGTTGGGTGCCAAGGGTGTTTAAT-TTCTCCA--TGTATGAAGAAATTTGACTTG
                ***. *:.**:.***: * * *: .**  *.* **  : *******::  ** * :**
```

```
Hm1-B73       CAAATGAT----TCACTCGAAAAAACAAAAGTATCAA---AATCATAGTATTTTCTCTCT
Hm1A          CTTGAGATTTAAACTCACCTAATTACATTTAATTCATATCAATCCAAGTGGATTG-----
Hm1-Va35      CTTGAGATTTAAACTCACCTAATTACATTTAATTCATATCAATCCAAGTGGATTG-----
Hm1-Pr1       CTTGAGATTTAAACTCACCTAATTACATTTAATTCATATCAATCCAAGTGGATTG-----
hm1-Pr        CTTGAGATTTAAACTTACCTAATTACATTTAATTCATATCAATCCAAGTGGATTG-----
              *::.:***    :*: :* :**::***:::.::***:   ****.:***. :**

Hm1-B73       CTAAAATTCCAAAAATATTTACATCTAAAGTTGATGTCTTGGCCTCCCGCGCTGTTTTA -241 from TSS
Hm1A          AGAGCTAACCA------ACAAAACCCTAAAGTTGATGTCTTGACCTCCCGTGCAGTTTTA
Hm1-Va35      AGAGCTAACCA------ACAAAACCCTAAAGTTGATGTCTTGACCTCCCGTGCAGTTTTA
Hm1-Pr1       AGAGCTAACCA------ACAAAACCCTAAAGTTGATGTCTTGACCTCCCGTGCAGTTTTA
hm1-Pr        AGAGCTAACCA------ACAAAACCCTAAAGTTGATGTCTTGACCTCCCGTGCAGTTTTA
              . *..:::***      : ::*.. ****************.******* **:******

Hm1-B73       CCTTCCTCCTCTGGACACCAACCACAGGCCGACAACCGAGTAAGCCGGTCAAT-TTTGGT -181 from TSS
Hm1A          CCTTCCTCCTCTGGACACCAACCACAGGCCGACAACCGAGTAAGCCGGTCAAATTTTGGT
Hm1-Va35      CCTTCCTCCTCTGGACACCAACCACAGGCCGACAACCGAGTAAGCCGGTCAAATTTTGGT
Hm1-Pr1       CCTTCCTCCTCTGGACACCAACCACAGGCCGACAACCGAGTAAGCCGGTCAAATTTTGGT
hm1-Pr        CCTTCCTCCTCTGGACACCAACCACAGGCCGACAACCGAGTAAGCCGGTCAAATTTTGGT
              ****************************************************: ******

Hm1-B73       ATCCTGCTCATGACTC-----------------------ATATCAGGCGGTAGCCGAGCA
Hm1A          ATCCTGCTCATGACTCATGATTATATTTAAACTACACACATATCAGGCGGCAGCCGAG--
Hm1-Va35      ATCCTGCTCATGACTCATGATTATATTTAAACTACACACATATCAGGCGGCAGCCGAG--
Hm1-Pr1       ATCCTGCTCATGACTCATGATTATATTTAAACTACACACATATCAGGCGGCAGCCGAG--
hm1-Pr        ATCCTGCTCATGACTCATGATTATATTTAAACTACACACATATCAGGCGGCAGCCGAG--
              ****************                       *********** *******

Hm1-B73       GCCGCCCAGCTTTCTCATGCCAGAGCAAACCCATAGGTCCAGTCCAAATCCAATCCCTGT
Hm1A          -CCGCCCATCTTTCTCATGCCAGAGCAAACCCATAGGTCCAGTCCAAATCCAATCCCTGT
Hm1-Va35      -CCGCCCATCTTTCTCATGCCAGAGCAAACCCATAGGTCCAGTCCAAATCCAATCCCTGT
Hm1-Pr1       -CCGCCCATCTTTCTCATGCCAGAGCAAACCCATAGGTCCAGTCCAAATCCAATCCCTGT
hm1-Pr        -CCGCCCATCTTTCTCATGCCAGAGCAAACCCATAGGTCCAGTCCAAATCCAATCCCTGT
               ******* ***************************************************
```

```
Hm1-B73        TGCCATCAGAATTTCAGGGGCAGCCATG
Hm1A           TGCCATCAGAATTTCAGGGGCAGCCATG
Hm1-Va35       TGCCATCAGAATTTCAGGGGCAGCCATG
Hm1-Pr1        TGCCATCAGAATTTCAGGGGCAGCCATG
hm1-Pr         TGCCATCAGAATTTCAGGGGCAGCCATG
               ****************************
```
