# Supplementary material for: Adult plant resistance in maize to northern leaf spot is a feature of partial loss-of-function alleles of Hm1
Source: PLoS Pathog. 2018 Oct 17;14(10):e1007356. doi: 10.1371/journal.ppat.1007356 (PMC6205646; doi:10.1371/journal.ppat.1007356)
Supplement: S2 Fig — Sequence comparison of HM1A with HM1 from the resistant maize inbreds (B73, Va35, W22, and Pr1), maize cultivars (Enano and Pira), HM1 homologs [sorghum (Sorghum bicolor), rice (Oryza sativa), and barley (Hordeum vulgare)], and maize dihydroflavonol 4-reductase (DFR). Among the five amino acid substitutions present in HM1A (highlighted in red and bold), the L116 residue was a conserved in all Hm1 alleles and orthologs. (PDF) [file ppat.1007356.s002.pdf]

S2 Fig. HM1 peptide sequence comparison of *hm1* alleles. Sequence comparison of HM1^{A} with HM1 from the resistant maize inbreds (B73, Va35, W22, and Pr1), maize cultivars (Enano and Pira), HM1 homologs [sorghum (*Sorghum bicolor*), rice (*Oryza sativa*), and barley (*Hordeum vulgare*)], and maize dihydroflavonol 4-reductase (DFR). Among the five amino acid substitutions present in HM1^{A} (highlighted in red and bold), the L116 residue was a conserved in all *Hm1* alleles and orthologs.

```
HM1-B73          -------MAEKESNGVRVCVTGGAGFIGSWLVRKLLEKGYTVHATLRNTGDEAKAGLLRR
HM1A             -------MAEKESNGVRVCVTGGAGFIGSWLVRKLLEKGYTVHATLRNTGDEAKAGLLRR
HM1-Va35         -------MAEKESNGVRVCVTGGAGFIGSWLVRKLLEKGYTVHATLRNTGDEAKAGLLRR
HM1-Pr1          -------MAEKESNGVRVCVTGGAGFIGSWLVRKLLEKGYTVHATLRNTGDEAKAGLLRR
HM1-W22          -------MAEKESNGVRVCVTGGAGFIGSWLVRKLLEKGYTVHATLRNTGDEAKAGLLRR
HM1-Enano        --------------------------------------------------DEAKAGLLRR
HM1-Pira         --------------------------------------------------DEAKAGLLRR
HM1-Sorghum      -------MAENESSGVRVCVTGGAGFIGSWLVKKLLEKGYTVHATLRNTGDEEKAGLLRR
HM1-Rice         MAEEGR-SGGVAGDGVRVCVTGGAGFIASWLVKKLLERGCIVHATLRSMGDEEKAGLLRR
HM1-Barley       MAEEGKGTGSNGGRGVRACVTGGAGFIGSWLVRKLLEAGYTVHATLRSIGDEGKAGLLRG
DFR-Maize        ----------MAGGGATVCVTGAGGFIASWVVKLLLSRGYTVHGTVRHLSDEKT-GHLKR
                                                              S99Y       D110Y
HM1-B73          LVPGAA--ERLRLFQADLFDAATFAPAIAGCQFVFLVATPFGLDSAGSQYKSTAEAVVDA
HM1A             LVPGAA--ERLRLFQADLFDAATFAPAIAGCQFVFLVATPFGLDSAGYQYKSTAEAVVYA
HM1-Va35         LVPGAA--ERLRLFQADLFDAATFAPAIAGCQFVFLVATPFGLDSAGYQYKSTAEAVVDA
HM1-Pr1          LVPGAA--ERLRLFQADLFDAATFAPAIAGCQFVFLVATPFGLDSAGYQYKSTAEAVVDA
HM1-W22          LVPGAA--ERLRLFQADLFDAATFAPAIAGCQFVFLVATPFGLDSAGSQYKSTAEAVVDA
HM1-Enano        LVPGAA--ERLRLFQADLFYAATFAPAIDGCQFVFLVATPFGLDSAGSQYKSTAEAVVYA
HM1-Pira         LVPGAA--ERLRLFQADLFDAATFAPAIAGCQFVFLVATPFGLDSAGYQYKSTAEAVMYA
HM1-Sorghum      LVPGAA--ERLRLFDADLFDAATFAPAIAGCQFVFLVATPFGLQDAGSKYKSTAEAVVDA
HM1-Rice         LVPGAA--ERLRLFEADLFDAATFAPAIAGCQFVFLIATPYGLEASNSKYKNTADAAVDA
HM1-Barley       LVPGGAPPERLVLFEADIYDAASFAPAIAGCHFVFLVANPSAHE-PASKYKTSAEAAADA
DFR-Maize        LENA---AGNLRIFKADLLDYDAMAAAVVGCQGVFHVATPVPSEDLTDPELQMLGPAVTG
```

L116H

HM1-B73 VRAI**L**RQCEESRTVKRVIHTASVAAASPLLEEE-VSASGVGYRDFIDESCWTSLNVDYPL
HM1A VRAI**H**RQCEESRTVKRVIHTASVAAASPLLEEE-VSASGVGYRDFIDESCWTSLNVDYPL
HM1-Va35 VRAI**L**RQCEESRTVKRVIHTASVAAASPLLEEE-VSASGVGYRDFIDESCWTSLKVNYPL
HM1-Pr1 VRAI**L**RQCEESRTVKRVIHTASVAAASPLLEEE-VSASGVGYRDFIDESCWTSLNVDYPL
HM1-W22 VHAI**L**RQCEESRTVKRVIHTASVAAASPLLEEE-VPASGVGYRDFIDESCWTSLNVDYPL
HM1-Enano VRAI**L**RQCEESRTVKRVIHTASVAAASPLLEEE-VSASGVGYRDFIDESCWTSLNVDYPL
HM1-Pira VRAI**L**RQCEESRTVKRVIHTASVAAASPLLEEE-VSASGVGYRDFIDESCWTSLNVDYPL
HM1-Sorghum VRAI**L**RQCEESRTVKRVIHTASVSAASPLKEEE-VS-GVIGYREFISESCWTSPDVDYPL
HM1-Rice VREI**L**RQCAESKTVKRVIHTASISTASPLIDVPGAGVGAAGYRDFIDESCWTPLDVDYPL
HM1-Barley VRVI**L**RLCAESRTVKRVIHTASVTAASPLTKSSS--AAAAVYGDFISESCWTLLDVNYPL
DFR-Maize TTNV**L**KA-ASSANVQRVVVVSSMVAVE-------ISPKDWPEGKVRDERCWSDREFCRSI

S191N

HM1-B73 RSAHFDKYILSKLRSEQELL**S**YNGGESPAFEVVTLPLGLVAGDTVLGRAPETVESAVAPV
HM1A RSAHFDKYILSKLRSEQELL**N**YNGGESPAFEVVTLPLGLVAGDTVLGRAPETVESAVAPV
HM1-Va35 RSAHFDKYILSKLRSEQELL**S**YNGGESPAFEVVTLPLGLVAGDTVLGRAPETVESAVAPV
HM1-Pr1 RSAHFDKYILSKLRSEQELL**S**YNGGESPAFEVVTLPLGLVAGDTVLGRAPETVESAVAPV
HM1-W22 RSAHFDKYILSKLQSEQELL**S**YNNGESPAFEVVTLPLGLVAGDTVLGRAPETVESAVAPV
HM1-Enano RSAHFDKYILSKLRSEQELL**N**YNGGESPAFEVVTLPLGLVAGDTVLGCASETVESAVAPV
HM1-Pira RSAHFDKYILSKLRSEQELL**N**YNGSESPAFEVVTLPLGLVAGDTVLGRAPETVESAVAPV
HM1-Sorghum RSAHFDKYILSKVQSEQELL**S**YNGGESPAFEVVTLALGLVAGDTVLGRVPETVESAVSPV
HM1-Rice RSAHFDKYVLSKMMSEKELL**G**YNDGEGRAFEVVTLPCGLVAGDTVLGRAPETLENAVSPV
HM1-Barley RSVHFDKYIESKVLSEKELL**S**YNDGENPAFEVVTLPLGLVGGNTVLGYLPETMESVVAPV
DFR-Maize E----SWYPVAKIISEEAAL**A**YG--QQTGLDVVTINPGLVFGPMLQPTVNTTIQFLI---

L240P

HM1-B73 SRSEPCFGL**L**RILQQLLGSLPLVHVDDVCDALVFCMERRPSVAGRFLCAAAYPTIHDVVA
HM1A SRSEPCFGL**P**RILQQLLGSLPLVHVDDVCDALVFCMERRPSVAGRFLCAAAYPTIHDVVA
HM1-Va35 SRSEPYFGL**P**RILQQLLGSLPLVHVDDVCDALVFCMERRRSVAGRFLCAAAYPTIHDVVA
HM1-Pr1 SRSEPCFGL**P**RILQQLLGSLPLVHVDDVCDALVFCMERRPSVAGRFLCAAAYPTIHDMVA
HM1-W22 SRSEPYFGL**L**RILQQLLGSLPLVHVDDVCDALVFCMERRPSVAGRFLCAAAYPTIHDVVA
HM1-Enano SRSEPYFGL**L**RILQQLLGSLPLVHVDDVCDALVFCMERRPSVAGRFLCAAA---------
HM1-Pira SRSEPCFGL**P**RILQQLLGSLPLVHVDDVCDALVFCMERRPSVAGRFLCAAA---------
HM1-Sorghum SRNEAYFGL**P**RILQQLLGSLPLVHVDDVCDALIFCMERRHSIAGRFLCAAAYPTIHDVVG
HM1-Rice SRNEPSFAF**L**RLLQRLVGSVPLVHADDVCDALVFCMDQ-PSLAGRFLCSAAYPTIHDIVE
HM1-Barley TKQEPCFML**P**RILQRLLGSVPLVHVDDVCAALIFCMEQ-PALSGRFLCAAAYPTIHDILD
DFR-Maize -----YFLK**G**GPDPVKNKLWHIVDVRDVADAMLLLYEV-PEATGRHICAPHVISARDLLD

HM1-B73	HYASKFPHLDILKET-EAVATV---RPARDRLGELGFKYKYGMEEILDSSVACAARLGSL
HM1A	HYASKFPHLDILKET-EAVATV---RPARDRLGELGFKYKYGMEEILDSSVACAARLGSL
HM1-Va35	HYASKFPHLDILKET-EAVATV---RPARDRLGELGFKYKYGMEEILDSSVACAARLGSL
HM1-Pr1	HYASKFPHLDILKET-EAVATV---RPARDRLGELGFKYKYGMEEILDSSVACAARLGSL
HM1-W22	HYASKFPHLDILKETTEAVATV---RPARDRLGELGFKYKYGMEEILDSSVACAARLGSL
HM1-Enano	--
HM1-Pira	--
HM1-Sorghum	HYARKFPHLDILKET-EAVARV---QPDGDRLGELGFVYKYGIEEILDSSVACAARLGCL
HM1-Rice	HFAAKYPHLDVLKEPEREVARV---QPAADKLGELGFRYKYGMEEILDGSVGCAARLGYI
HM1-Barley	HYGSKYPHLDLLRET-DEVARV---QPDKNKLGELGFEYKYGLKEILDESIDCAVRLGSL
DFR-Maize	LLKSMYPDYPCIANE-SILDRDHPAPMTSDKLKKIGWSCRP-LEETIVDTVECCLRAGFL

HM1-B73	DASKLGLQKG--------
HM1A	DASKLGLQKG--------
HM1-Va35	DASKLGLQKG--------
HM1-Pr1	DASKLGLQKA--------
HM1-W22	DGSKLGLQKG--------
HM1-Enano	------------------
HM1-Pira	------------------
HM1-Sorghum	DATKLSMQKG--------
HM1-Rice	DAAKLRPQEG--------
HM1-Barley	DASKLIVQQG--------
DFR-Maize	DDVGGETSCRFPPLLNQI
