# Supplementary material for: Adult plant resistance in maize to northern leaf spot is a feature of partial loss-of-function alleles of Hm1
Source: PLoS Pathog. 2018 Oct 17;14(10):e1007356. doi: 10.1371/journal.ppat.1007356 (PMC6205646; doi:10.1371/journal.ppat.1007356)
Supplement: S3 Fig — (A) Ethyl methanesulfonate (EMS) mutagenesis caused a G to A substitution at the junction of exon3/intron3. (B) Predicted protein sequence of hm1-2 is 176 amino acids similar to HM1-B73 and the mutation in the intron is predicted to generate 26 different amino acids before resulting in a truncated protein. (PDF) [file ppat.1007356.s003.pdf]

S3 Fig. Null allele *hm1-2* identified in B73-EMS M_2 population. (A) Ethyl methanesulfonate (EMS) mutagenesis caused a G to A substitution at the junction of exon3/intron3. (B) Predicted protein sequence of *hm1-2* is 176 amino acids similar to $HM1^{B73}$ and the mutation in the intron is predicted to generate 26 different amino acids before resulting in a truncated protein.

A

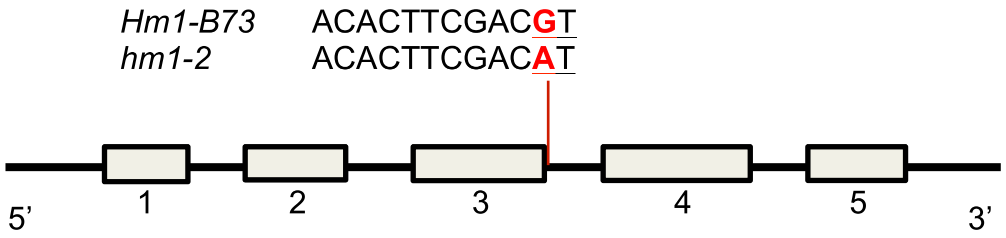

B

```
Hm1-B73       MAEKESNGVRVCVTGGAGFIGSWLVRKLLEKGYTVHATLRNTGDEAKAGLLRRLVPGAAE  60
hm1-2         MAEKESNGVRVCVTGGAGFIGSWLVRKLLEKGYTVHATLRNTGDEAKAGLLRRLVPGAAE  60
              ************************************************************

Hm1-B73       RLRLFQADLFDAATFAPAIAGCQFVFLVATPFGLDSAGSQYKSTAEAVVDAVRAILRQCE  120
hm1-2         RLRLFQADLFDAATFAPAIAGCQFVFLVATPFGLDSAGSQYKSTAEAVVDAVRAILRQCE  120
              ************************************************************

Hm1-B73       ESRTVKRVIHTASVAAASPLLEEEVSASGVGYRDFIDESCWTSLNVDYPLRSAHFDKYIL  180
hm1-2         ESRTVKRVIHTASVAAASPLLEEEVSASGVGYRDFIDESCWTSLNVDYPLRSAHFDISSI  180
              ********************************************************   :

Hm1-B73       SK-------LRSEQELLSYNGGESPAFEVVTLPLGLVAGDTVLGRAPETVESAVAPVSRS  233
hm1-2         QAKLLLISELERLITLIFFS----------------------------------------  200
              .        *.    *: :.

Hm1-B73       EPCFGLLRILQQLLGSLPLVHVDDVCDALVFCMERRPSVAGRFLCAAAYPTIHDVVAHYA  293
hm1-2         ------------------------------------------------------------  200

Hm1-B73       SKFPHLDILKETEAVATVRPARDRLGELGFKYKYGMEEILDSSVACAARLGSLDASKLGL  353
hm1-2         ------------------------------------------------------------  200

Hm1-B73       QKG  356
hm1-2         ---  200
```
