# Supplementary material for: Adult plant resistance in maize to northern leaf spot is a feature of partial loss-of-function alleles of Hm1
Source: PLoS Pathog. 2018 Oct 17;14(10):e1007356. doi: 10.1371/journal.ppat.1007356 (PMC6205646; doi:10.1371/journal.ppat.1007356)
Supplement: S4 Fig — (A) Hm1B73 plants were resistant at all stages of plant development. Two novel APR alleles, Hm1-3 (B) and Hm1-4 (C), generated by targeted EMS mutagenesis were susceptible as seedlings (week-3) and became resistant to CCR1 at week-7. A novel null allele, hm1-5 (D) remained susceptible throughout the age of the plant. (PDF) [file ppat.1007356.s004.pdf]

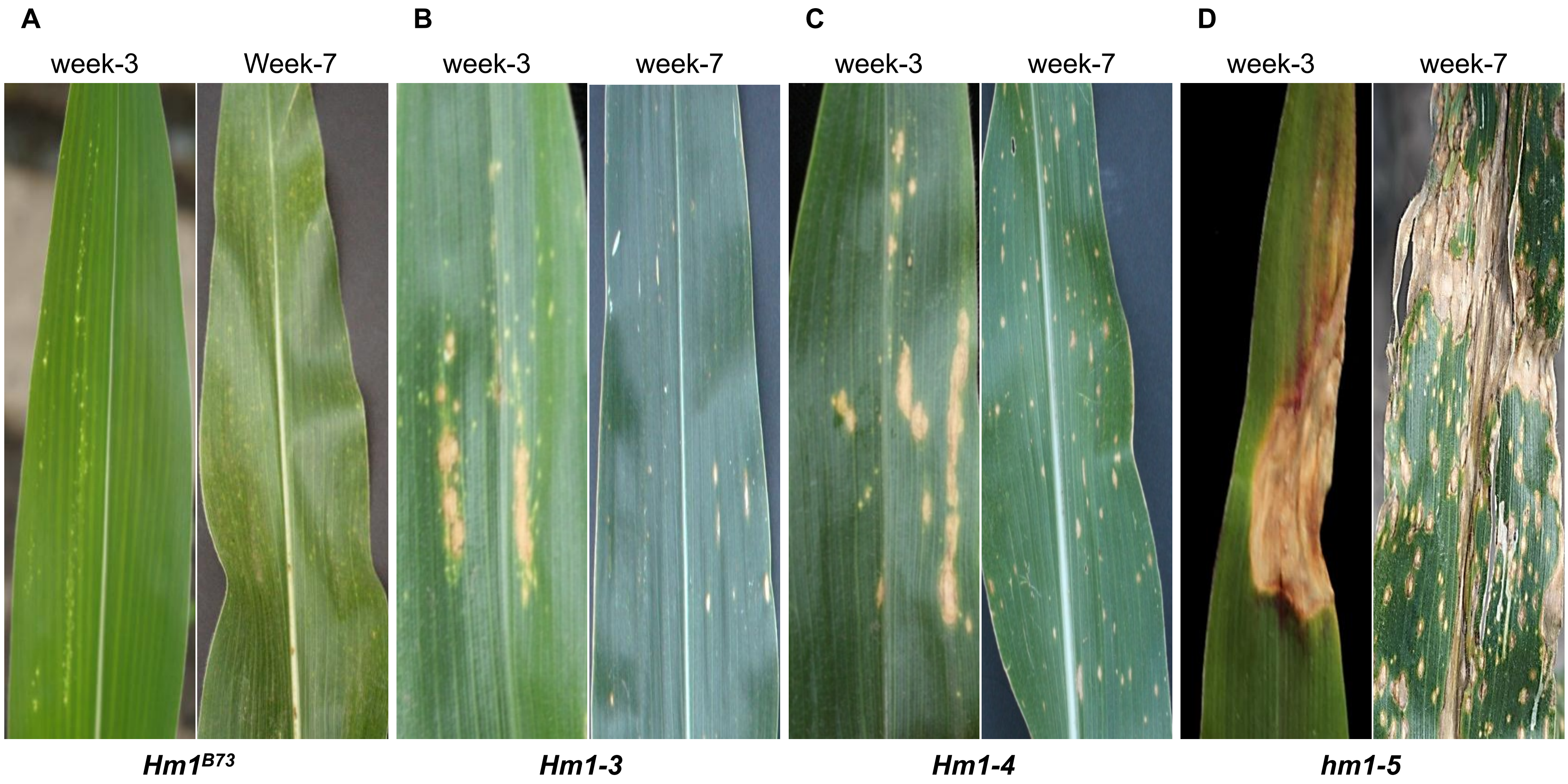

S4 Fig. Disease response of two novel APR alleles generated using EMS mutagenesis.

(A) *Hm1*^{B73} plants were resistant at all stages of plant development. Two novel APR alleles, *Hm1-3* (B) and *Hm1-4* (C), generated by targeted EMS mutagenesis were susceptible as seedlings (week-3) and became resistant to CCR1 at week-7. A novel null allele, *hm1-5* (D) remained susceptible throughout the age of the plant.
