# Supplementary material for: Adult plant resistance in maize to northern leaf spot is a feature of partial loss-of-function alleles of Hm1
Source: PLoS Pathog. 2018 Oct 17;14(10):e1007356. doi: 10.1371/journal.ppat.1007356 (PMC6205646; doi:10.1371/journal.ppat.1007356)
Supplement: S5 Fig — Four primer pairs that preferentially amplified overlapping sequences of the Hm1B73 allele over the hm1Pr allele from heterozygous M1 plants generated using the targeted EMS mutagenesis screen. Grey boxes represent the exons while the black lines between the exons represent the introns. Different primer pairs used to amplify Hm1B73 are marked using different colors with the amplicon size listed above each fragment. (PDF) [file ppat.1007356.s005.pdf]

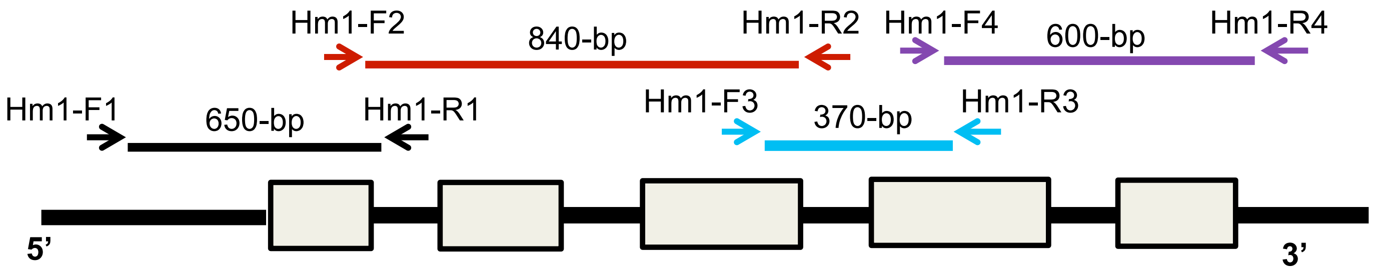

S5 Fig. Overlapping primers to amplify *Hm1*^{B73} from novel *Hm1* mutants.

Four primer pairs that preferentially amplified overlapping sequences of the *Hm1*^{B73} allele over the *hm1*^{Pr} allele from heterozygous M_1 plants generated using the targeted EMS mutagenesis screen. Grey boxes represent the exons while the black lines between the exons represent the introns. Different primer pairs used to amplify *Hm1*^{B73} are marked using different colors with the amplicon size listed above each fragment.
