# Supplementary material for: Adult plant resistance in maize to northern leaf spot is a feature of partial loss-of-function alleles of Hm1
Source: PLoS Pathog. 2018 Oct 17;14(10):e1007356. doi: 10.1371/journal.ppat.1007356 (PMC6205646; doi:10.1371/journal.ppat.1007356)
Supplement: S1 Table — Using PCR-based markers, APR alleles were introgressed into B73 genetic background. (PDF) [file ppat.1007356.s006.pdf]

S1 Table. Molecular marker (primer) combinations used to differentiate APR alleles from wild-type *Hm1*. Using PCR-based markers, APR alleles were introgressed into B73 genetic background.

**Project**	**Forward primer**	**Reverse primer**
**Primers for linkage analysis**		
Genetic cross: (P8 × Pr) × Pr	5'–GCCCACCAAGGTCCAGC–3'	5'–GATGTCGAGGTGAGGGAAC–3'
Differentiate *Hm1A* from *hm1* (256-bp insertion in exon4)	5'– CCGGCGTTCGAGGTGGTGA–3'	5'–GATGTCGAGGTGAGGGAAC–3'
Differentiate Hm1A and $Hm1^{Pr1}$ allele	5'–TACGCGGTGCGCGCGATCCA–3'	5'–CAGGCGTTCCAGTTCAGAA–3'
	5'– GACGCGGTGCGCGCGATCCT–3'	5'– CAGGCGTTCCAGTTCAGAA–3'
Hm1A introgressed into B73	5'–GAGGTGAGGTACCCTGGTCC–3'	5'– CAGACGCACCGGTGTTCCGC – 3'
Differentiate Hm1A from Hm1	5'–TACGCGGTGCGCGCGATCCA–3'	5'–CAGGCGTTCCAGTTCAGAA–3'
Differentiate novel B73 null allele (hm1-2) from Hm1	5'–TCCGAAGCGCACACTTCGACG–3'	5'–GATGTCGAGGTGAGGGAAC–3'
Differentiate Hm1A from Hm1-B73	5'–GAGGTGAGGTACCCTGGTCC–3'	5'– CAGACGCACCGGTGTTCCGC – 3'
	5'–GACCGCTAATGCTTGTTTAGACC–3'	5'–GCGTGTTTCGCCTTTCGCGACG –3'
Hm1-3 allele from Hm1-B73: CAPs marker - NlaII	5' GGCGAAACACGCCAAAGGCGA 3'	5' TTCTGA ACTGGAACGCCTG 3'
Hm1-4 allele from Hm1-B73: CAPs marker - NlaIII	5' GCTCAACGTTGACTATCCTCTCCG 3'	5' CGCTGGGTGCACGTGGACGACG 3'
Hm1-5 allele from Hm1-B73: CAPs marker - KpnI	5' GGCGAAACACGCCAAAGGCGA 3'	5' TTCTGA ACTGGAACGCCTG 3'
Amplify *Hm1* over hm1 from novel EMS mutants	5' GACCGCTAATGCTTGTTTAGACC 3'	5' GCGTCGCGAAAGGCGAAACACGC 3'
	5' GGCGAAACACGCCAAAGGCGA 3'	5' TTCTGA ACTGGAACGCCTG 3'
	5' GCTCAACGTTGACTATCCTCTCCG 3'	5' CGCTGGGTGCACGTGGACGACG 3'
	5' GCTCCTGAGCTACAACGGCGGC 3'	5' GGGGAACTTGGATTGTTCGCT 3'
Cloning of $Hm1^{A}$ cDNA	5'-ATGGCCGAAAAGGAGAGCAACG-3'	5'-TTATCCTTTCTGTAGGCCGAG-3'
